# Occupational therapy: The key to unlocking locked-up occupations during the COVID-19 pandemic

**DOI:** 10.12688/wellcomeopenres.16089.1

**Published:** 2020-07-01

**Authors:** Sureshkumar Kamalakannan, Stuti Chakraborty

**Affiliations:** 1 SACDIR - Indian Institute of Public Health, Public Health Foundation of India, Hyderabad, Telangana, 500033, India; 2 ICED Clinical Research Department, London School of Hygiene & Tropical Medicine, London, England, WC1E 7HT, UK; 3 India Alliance (DBT - Wellcome Trust), Hyderabad, India; 4 Occupational Therapy Rehabilitation Institute, Christian Medical College, Vellore, Tamil Nadu, 632002, India

**Keywords:** Occupational Science, Occupational Therapy, Covid-19, Pandemic, Lockdown Coronavirus, Activity Analysis, Occupations

## Abstract

Occupations refer to the everyday activities that people do as individuals, in families and with communities to occupy time and bring meaning and purpose to life. It is not always limited to just paid employment. Occupations of the global population have been adversely affected in one way or the other because of this COVID-19 pandemic. Four different key sects of occupations were majorly affected. These are the occupations of those who are or were COVID-positive, occupations of healthy individuals affected by COVID-19/lockdown, occupations of the population highly susceptible and vulnerable of contracting COVID-19 and occupations having a direct impact on global market, supply chain or economy. These occupations were locked up due to the pandemic lockdown.

Occupational therapists can scientifically analyse occupations and help formulate exit strategies for the lockdown. They are experts who understand and study the different ways of measuring participation in occupation to develop innovative strategies and therapeutic interventions to facilitate individuals’ engagement in occupations. They can unravel the pragmatic strategies for preventing transmission (physical distancing, hand hygiene, personal protective equipment usage and decontamination) despite engaging in occupations safely and effectively. Nourishing this niche and essential science is pertinent, not just in this pandemic context but also against a backdrop of health and social care research, policy, practice and education for the future.

The word “occupation” in layman’s terms refers to any activity that an individual meaningfully engages with, in his everyday living ^1^. Scientifically, “occupations refer to the everyday activities that people do as individuals, in families and with communities to occupy time and bring meaning and purpose to life. Occupations include things people need to, want to and are expected to do” ^2^. Both ways, the term covers everything that an individual does in his/her life from womb to tomb. However this term is globally misunderstood to be related only to paid employment and having socio-cultural dimensions of normality ^3^. In simple terms some occupations are considered appropriate in certain cultures and not in others ^4^.

The recent coronavirus disease 2019 (COVID-19) pandemic has made a devastating impact disrupting global equilibrium of sustainable development and meaningful occupations that define the lives of millions of people worldwide ^5^. Researchers, scientists, policy-makers and politicians around the world have been left overwhelmed with the number of deaths due to COVID-19. However, the attention given to affected individuals’ occupations as defined above has remained absolutely meagre in comparison ^6^.

Occupations of the global population have been adversely affected because of this pandemic ^7^. Children are unable to play in the park with their peers or study at school, adults are unable to cope with adjusting their schedules while working from home, with home schooling and children around. A significant proportion of this occupational struggle can be found especially in low- and middle-income countries where several of the poorest have lost their only source of income, while such countries continue to remain sparse in terms of resources when compared to high-income countries ^8^. The elderly, being considered more vulnerable are being shielded from what they might consider as meaningful occupations like visiting their grandchildren, engaging in social activities and leisure. Overall, the pandemic and onset of the lockdown globally has not just locked up all of us in our homes, but also confined and restricted us in carrying out our occupations ^9^.

A deeper reflection of the pandemic and lockdown unravels four different key sects of occupations that have been eyed upon by politicians, expert scientists and activists to be rescued. These are the occupations of those who are or were COVID-positive, occupations of healthy individuals affected by COVID-19/lockdown, occupations of the population highly susceptible and vulnerable of contracting COVID-19 and occupations having a direct impact on global markers, supply chain or economies. The impact that each sect of occupations has gone through and continues to go through is very different. Similarly, the implications as well; some occupations have financial, some health, some political while others seem to have policy implications. However, a potential enabler to the derived solutions that stakeholders across multiple levels have failed to identify is to seek the key to unlock these occupations ^10^.

The key is the science of occupation, and the experts who know how to use the key to unlock occupations are highly trained healthcare professionals—occupational therapists—who have immense expertise in field of occupational science and therapy ^11^. Occupational science is a science related to studying occupations in which humans participate. Occupational science and therapy often focus on specific populations who have unique challenges to participate in meaningful occupations. Occupational therapists are experts in this field who understand and study the different ways of measuring participation to develop innovative strategies and therapeutic interventions to facilitate individuals’ engagement in occupations. They also study different ways of measuring participation to develop innovative interventions that enable occupational engagement, thereby preventing the negative effects of diseases and disability and ultimately promoting the positive impact of participation in occupations on an individual’s health and well-being ^12^.

Occupational therapists use a fundamental and powerful tool to help individuals re-engage in occupations, known as activity analysis ^13^. It is the process of identifying inherent properties in any given occupation as well as the skills and abilities to complete it. Occupational therapists deconstruct a single occupation into many components to see the best fit of individual needs, capabilities, activity characteristics in the actual environment or context of their survival, thereby optimising successful performance of a particular occupation when an individual cannot meaningfully engage in it ^14^.

The current global pandemic and lockdown has propelled scientists, researchers, politicians, policymakers and various other stakeholders to come up with immediate and sustainable long-term solutions. Recommendations and guidelines from various global and national-level stakeholders/organisations on the pandemic and lockdown have been looking at various targeted, multi-phased strategies to unlock occupations. Though “Exit strategy” may be the term used by these stakeholders for that purpose, these interventions indirectly target unlocking occupations from the pandemic lockdown, with the aim of helping people across ages engage in meaningful occupations enable sustainable global development ^10, 15^.

Occupational therapists can scientifically analyse occupations and crack the code to such exit strategies. They can do so by:

1. Assisting in the process of classifying occupations that are at higher, moderate and lower risk for infection transmission, prioritising those occupations that could be unlocked for specific social, economic or well-being purposes as required.
2. Providing scientific knowledge and support to reduce, prevent and control transmission of COVID-19 while engaging in any occupation. Occupational therapists can analyse occupations those are at higher risk for spread and transmission such as accessing public transport facilities including bus, train, ferry and flights and they can identify potential solutions to safely restart these services with utmost precautions to prevent transmission. Occupational therapists can provide pragmatic solutions to ensure physical distancing, hand hygiene and use of personal protective equipment (PPE) while individuals adapt or modify their occupations within their usual environments ^16^. Given their skills for environmental adaptations and modifications, creative solutions put forward by occupational therapists could be potentially feasible to implement while exiting the lockdown ^17^.
3. 3. Having an imperative role in synergising public health efforts focused on safe practices in multiple contexts, such as hospitals, care homes, rehabilitation centres and special schools where they are employed . Since occupational therapists are professionally trained in the field of healthcare practice and research, they also have a key role to play on the frontlines by contributing towards improving patient outcomes in the management of COVID-19, alongside physicians, nurses and other allied health professionals ^18^.
4. Exploring options for remediation and adaptation in case of complicated occupations ^19^. For example: In order to restart schooling services, occupational therapists can identify and analyse various occupations of the staff involved (teachers, administrators, support staff and their requirements within the environmental context) in order to provide strategies for ensuring safe and effective schooling.
5. Remediating issues related to adopting a common strategy like physical distancing for each occupation. The above example of a schoolteacher can be taken again: occupational therapists can provide guidance on how the teacher can ensure physical distancing while engaging in her/his primary task of taking a class as well as other secondary tasks such as meeting colleagues or supervising children participating in recess.

Occupational therapists can be of immense help in the rapid implementation of various public health solutions for behaviour change aiming to prevent and control the rate of infection spread among individuals globally ^20^. A plethora of assessment techniques used by occupational therapists, such as work flow analysis, assessing workspace design, performing risk assessments for specific kinds of activities demanded by each occupation could help understand the ways in which services can ensure safety and avoid risk of transmission when they restart ^21^. Though current behaviours are being moulded towards sustaining the “NEW NORMAL”, whether the new normal life has meaningful engagement of individuals for safe and healthy well-being is a million-dollar question.

It is high and critical time that every stakeholder involved in combating COVID-19 especially in low- and middle-income countries appreciate the under-represented and untapped scientific expertise that occupational therapists possess. Evidence suggests that there are only 0.03 occupational therapists per 100,000 people globally ^22^. Although a miniscule proportion of clinician scientists, they are not included in scientific or public health cadre for disease prevention, health promotion and rehabilitation, particularly in the health systems of the LMICs. Unlike high-income countries, this allied health workforce is not a part of the organized government health and social care system, even in globally emerging economies like China and India.

Occupational therapists therapeutically support persons with disabilities in general. Occupational therapy and science help people engage in meaningful occupations for their well-being. However, when we look at disability from a bio-psychosocial perspective, it is quite evident that the whole world is currently experiencing a temporary form of disability due to the pandemic. Nurturing this niche and essential science is pertinent, not just in the context of this pandemic but also against a backdrop of health and social care research, policy, practice and education for the future. 

## Data availability

No data are associated with this article.
