# Supplementary material for: Occupational therapy: The key to unlocking locked-up occupations during the COVID-19 pandemic
Source: Wellcome Open Res. 2020 Jul 1;5:153. [Version 1] doi: 10.12688/wellcomeopenres.16089.1 (PMC7388192; doi:10.12688/wellcomeopenres.16089.1)
Supplement: Peer review history

# Peer review: Reviewer response for version 1

**DOI:** 10.21956/wellcomeopenres.17654.r39541

**Published:** 2020-08-10

**Authors:** Lynette Mackenzie

**Affiliations:** 1 Occupational Therapy, University of Sydney, Sydney, NSW, Australia

Abstract: I do not think that occupations are merely about occupying time, but are about enabling people to do the things they need to do, have to do and want to do.

 The word "sects" is not helpful. I think you mean "areas" here.

 Vulnerable "to" is clearer.

 Not all occupations were locked down - I think you can say a substantial number of valued occupations were locked up or reduced. 

 OTs may not be able to help people exit the lockdown if it is a government requirement. However, they can assist people in adapting their occupations and devise ways to find purpose and meaning in everyday activities where the environment is a barrier.

 Nourishing the niche does not make much sense to me. I think you are talking about occupational therapists suing their skills in a new area of practice?  I'm sure other health professionals will assume they are contributing to preventing transmission!

 Content: Not sure I like the phrase "womb to tomb" - sounds like a colloquial amusing phrase rather than the serious point you are making.

 I think we are only just beginning to see the impact of recovery from COVID in terms of the occupations that are affected by the recovery process and the limitations in functioning that a lot of people in recovery experience.

 Again the term "sects" is used - would suggest "areas" instead. "Eyed upon" would be better replaced by "recognised by", and "rescued" is misleading - should be "addressed".

 Be careful about claiming "immense" expertise - expertise is sufficient. 

 What about other points such as assisting people who are forced into isolation by government requirements to improve their health and wellbeing in these circumstances, and assisting people recovery from a COVI infection who are experiencing fatigue, breathlessness and reduced capacity to engage in occupations?

 "It is a critical time" - sounds simpler. 

 Again "nurturing this niche" is not a good expression.

Does the article adequately reference differing views and opinions?

Yes

Are all factual statements correct, and are statements and arguments made adequately supported by citations?

Yes

Is the Open Letter written in accessible language?

Yes

Where applicable, are recommendations and next steps explained clearly for others to follow?

Yes

Is the rationale for the Open Letter provided in sufficient detail?

Yes

Reviewer Expertise:

Occupational therapy practice

I confirm that I have read this submission and believe that I have an appropriate level of expertise to confirm that it is of an acceptable scientific standard, however I have significant reservations, as outlined above.

# Peer review: Reviewer response for version 1

**DOI:** 10.21956/wellcomeopenres.17654.r39469

**Published:** 2020-07-28

**Authors:** Ashish S. Macaden

**Affiliations:** 1 Rehabilitation Medicine and Stroke, Raigmore Hospital, Inverness, UK; 2 University of Aberdeen, Centre for Rural Health, Inverness, UK

This is a timely proposal for OT to take on an innovative role in Covid-19. 

1. Differing views: The overlap with OT and the discipline of occupational health has not been addressed.
2. Accessible language: Their choice of words could be misinterpreted e.g. sects of professions (was it meant to be sets?) and the use of gendered words (layman, his or his/her). 
3. Recommendations: The ability of OT to take on this role without specialist training is not discussed. The role of OT in addressing common sequelae of COVID-19 (fatigue, de-conditioning) has not been discussed.

Does the article adequately reference differing views and opinions?

Partly

Are all factual statements correct, and are statements and arguments made adequately supported by citations?

Yes

Is the Open Letter written in accessible language?

Partly

Where applicable, are recommendations and next steps explained clearly for others to follow?

Partly

Is the rationale for the Open Letter provided in sufficient detail?

Yes

Reviewer Expertise:

Rehabilitation Medicine - Vocational rehabilitation, Gait analysis. Stroke - Prehospital ultrasound.

I confirm that I have read this submission and believe that I have an appropriate level of expertise to confirm that it is of an acceptable scientific standard, however I have significant reservations, as outlined above.

# Peer review: Reviewer response for version 1

**DOI:** 10.21956/wellcomeopenres.17654.r39354

**Published:** 2020-07-21

**Authors:** Lakshmanan Sethuraman

**Affiliations:** 1 Centre for Addiction Medicine, National Institute of Mental Health and Neurosciences, Bengaluru, Karnataka, India

Article highlights the need for use of the skill possessed by the occupational therapists especially in terms of analysing the occupation. Adaptations in relation to environmental context in specific to architectural modifications to control the transmission is the need of the hour. It would have been more appropriate if the authors add:

1. The changes in relation to student seating in their example of school. 
2. It is equally important for the occupational therapy practitioners use the opportunity to deliver in NEW NORMAL situations (while stressing on the need for nurturing this science).

Does the article adequately reference differing views and opinions?

Yes

Are all factual statements correct, and are statements and arguments made adequately supported by citations?

Yes

Is the Open Letter written in accessible language?

Yes

Where applicable, are recommendations and next steps explained clearly for others to follow?

Yes

Is the rationale for the Open Letter provided in sufficient detail?

Yes

Reviewer Expertise:

Occupational Therapy, Psychosocial intervention in Substance use, Aftercare in Addiction, Telementoring

I confirm that I have read this submission and believe that I have an appropriate level of expertise to confirm that it is of an acceptable scientific standard.

# Peer review: Reviewer response for version 1

**DOI:** 10.21956/wellcomeopenres.17654.r39540

**Published:** 2020-07-21

**Authors:** Karthik Mani

**Affiliations:** 1 National Board for Certification in Occupational Therapy, Gaithersburg, MD, USA; 2 Vijaya Occupational Therapy Centre, Chennai, India

The authors did a great job of highlighting how occupational therapists (OTs) may contribute to easing occupational restrictions secondary to Coronavirus Disease 2019 (COVID-19) and related lockdown. Further, they underscored the OTs expertise in activity analysis and environmental modification and its implications for the current crisis in a thoughtful manner. The following may be considered to improve clarity and readability, which may increase the impact of the idea being discussed in this open letter. 

 The first sentence of the article states that ‘occupation’ in layman’s terms refers to all meaningful daily living activities. However, this statement is misleading. As stated by the authors in the same paragraph, the word ‘occupation’, in layman’s terms, generally refers to paid employment. The authors may consider replacing ‘layman’s terms’ with ‘the context of occupational therapy’ to add clarity to the opening sentence.

 The sects of occupations described in this article do not appear to be distinct categories. For instance, ‘occupations having a direct impact on global market, supply chain or economy’ would also include ‘occupations of healthy individuals affected by COVID-19/lockdown’. The authors may elaborate more to improve clarity on how they perceive each of these sects as distinct categories. Further, they may underscore how OTs could contribute to each of this group more clearly.

 In paragraph 3, the authors stated that ‘adults are unable to cope with adjusting their schedules while working from home’, which, is an overgeneralized assumption. Do all adults working from home secondary to COVID-19 find it difficult to cope up? The authors may consider softening the tone by adding a qualifier such as ‘several’, ‘many’, etc. Alternatively, they may add citations to support this generalizing phrase.

 More focus on grammar, word conservation, use of punctuations, and consistent use of abbreviations is needed to improve clarity and readability. The authors may consider using readability statistics tools to assess and enhance readability. 

 Grammar - In a few places, the authors used past tenses. Given that the COVID-19 pandemic is still an ongoing crisis, past participle verb tenses would be more appropriate. Also, the authors may want to consider using conjunctions and articles effectively to improve the flow and readability.

 Word conservation: In the fourth paragraph, the authors used ‘has gone through’ and added ‘continues to go through’ in a sentence. The second part is unnecessary as ‘has gone through’ captures the meaning. Further, several sentences could be rephrased to conserve words as wordy sentences minimize the impact of the idea being discussed.

 Punctuations: Some long sentences could be punctuated with commas to improve readability. For example, in the penultimate paragraph, the phrase ‘especially in low- and middle-income countries’ could be placed between commas to break the sentence and improve readability.

 Abbreviations: ‘Occupational therapists’ was used throughout the paper. The authors may have used the abbreviation ‘OTs’ after the first use and used the abbreviation throughout the paper. The penultimate paragraph shows an abbreviation (LMICs referring to Low- and middle-income countries) which was not previously identified in the paper. If the authors decide not to use abbreviations, then they should plan to remain consistent throughout the paper.

 Typo: Point 3 under paragraph 8 has another ‘3’ (3. 3.), which needs to be deleted.

Does the article adequately reference differing views and opinions?

Partly

Are all factual statements correct, and are statements and arguments made adequately supported by citations?

Partly

Is the Open Letter written in accessible language?

Yes

Where applicable, are recommendations and next steps explained clearly for others to follow?

Yes

Is the rationale for the Open Letter provided in sufficient detail?

Yes

Reviewer Expertise:

Occupational therapy, professional issues, rehabilitation, survey research

I confirm that I have read this submission and believe that I have an appropriate level of expertise to confirm that it is of an acceptable scientific standard, however I have significant reservations, as outlined above.
